# The chronic intestinal ischemic disease in the elderly. Clinical - morphologic correlation study

**DOI:** 10.1186/1471-2318-11-S1-A18

**Published:** 2011-08-24

**Authors:** S Fratta, F Cardin, M Mosele, E Perissinotto, E M Inelmen, G Sergi, E Manzato, C Terranova

**Affiliations:** 1 Department of Medical and Surgical Sciences, Division of Geriatrics, University of Padua, Italy; 2 Department of Environmental Medicine and Public Health, University of Padua, Italy; 3 Geriatric Department, Division of Geriatric Surgery, University of Padua, Italy; 4 Toxicology and Antidoping Hospital University of Padua, Italy

## Background

It is a common clinical opinion that there is a mismatch between clinics and the morphological findings of mesenteric vascular district stenosis, anyway, very little experimental evidence exists relating to chronic intestinal ischemic pathology in the elderly and, in particular, it is not clear if there is a clinical picture emerging of ischemic pathology in the elderly.

The aim of the study is to evaluate the clinical and biohumoral presentation of the elderly with mesenteric vessels stenosis.

## Materials and methods

Patients over 64 years old that have undergone radiological examination of splanchnic vessels by AngioTC in the Azienda Ospedaliera of Padua and ULSS 16 between 2008 -2010 were included in this study. Patients who could not be interviewed or whose medical history could not be reconstructed through the hospital's archives, affected by primary renal failure, cirrhosis or neoplastic disease diagnosed within the past 5 years, who underwent splanchnic angioplastic surgery or intestinal resection, were excluded from the study. Patients with a clinical presentation of acute intestinal ischemia, were also excluded. These patients were then examined by the degree of vascular involvement to define two major groups: patients without vascular alterations or with monovascular involvement, and patients with multivascular involvement.

## Results

Ninety-nine patients weere studied 36 males and 63 females, with an average age of 76 years old, range 64-92. Between them 19 had, on examination, a multivascular alteration. The other 80 patients presented at the morphologic study a complete patency of mesenteric vessels, or a monovascular involvement. There were no significant differences between comorbidity and abdominal symptomatology, but there was a significant difference of age: the multivascular group was composed of older people (average age 78 years old), admissions and duration of hospitalization where higher and, from the hematochimic point of view, the multivascular group presented lower hemoglobin, MCV and albumin. Finally, in the group with multivascular alterations, the BMI was significantly lower and the use of benzodiazepines was higher than in the other group.

## Conclusions

From our data it emerges that elderly patients with vascular splanchnic alteration are subjected to major and longer hospitalizations, undergoing non specific treatment such as sedatives with benzodiazepines. This evidence, over and above characterizing a correspondence between seriousness of vascular damage and clinical course, could lead to the conclusion that vascular splanchnic alteration in the elderly is a not a broadly studied pathology and so is not completely managed within up-to-date experimental evidence.

